# Analysis of metabolomics associated with quality differences between room‐temperature‐ and low‐temperature‐stored litchi pulps

**DOI:** 10.1002/fsn3.1208

**Published:** 2019-09-30

**Authors:** Xiaomeng Guo, Tao Luo, Dongmei Han, Zhenxian Wu

**Affiliations:** ^1^ College of Horticulture South China Agricultural University/Guangdong Provincial Key Laboratory of Postharvest Science of Fruits and Vegetables/Engineering Research Center for Postharvest Technology of Horticultural Crops in South China Ministry of Education Guangzhou China; ^2^ Institute of Fruit Tree Research Guangdong Academy of Agricultural Sciences/Key Laboratory of Biology and Genetic Improvement of Horticultural Crops (South China) of Ministry of Agriculture Guangzhou China; ^3^ Guangdong Litchi Engineering Research Center/Key Laboratory of Biology and Genetic Improvement of Horticultural Crops‐South China Ministry of Agriculture Guangzhou China

**Keywords:** differentially expressed metabolites, litchi pulp, low‐temperature storage, quality, room‐temperature storage, UHPLC‐ESI‐QTOF‐MS/MS

## Abstract

Studies on how temperature affects the postharvest quality of litchi have focused mainly on pericarp browning but rarely on the metabolites in postharvest litchi pulp. In this study, the differences in respiration rates, total soluble solid content, and titratable acid content demonstrated that room and low temperatures have different effects on the quality of “Feizixiao” litchi pulp. UHPLC‐ESI‐QTOF‐MS/MS analysis was performed to compare the differentially expressed metabolites (DEMs) in litchi pulp after 8 days of storage at room temperature (RT‐8 d) with those in litchi pulp after 28 days of storage at low temperature (LT‐28 d). Nineteen carbohydrates (phosphohexoses, sorbitol, and mannose), fifteen acids, seven amino acids, nine energy metabolites and nucleotides, and six aliphatic and secondary metabolites were identified as common DEMs in RT‐8 d and LT‐28 d pulps. These findings indicated active fructose and mannose metabolism and increased catabolism of nicotinate, nicotinamide, alanine, aspartate, and glutamate. Four carbohydrates (mainly phosphohexoses), five acids, ten amino acids, three aliphatic and secondary metabolites, and one hormone were identified as unique DEMs in RT‐8 d pulp, the consumption of key metabolites in glycolysis and the tricarboxylic acid cycle, and accumulation of phenylalanine, tyrosine, and tryptophan. Active consumption of nucleotide metabolites and biosynthesis of aliphatics in LT‐28 d pulp were indicated by unique DEMs (eleven carbohydrates, four acids, seven amino acids, seven energy metabolites and nucleotides, and six aliphatic and secondary metabolites). These results provided an unambiguous metabolic fingerprint, thereby revealing how room and low temperatures differentially influenced the quality of litchi pulp.

## 1 INTRODUCTION

Litchi (*Litchi chinensis* Sonn.) is a perishable subtropical fruit that is widely cultivated in Asian countries. Litchi fruit is favored by consumers due to its attractive color, unique flavor, rich nutrition, and health benefits (Luo et al., 2019). Therefore, litchi is an important economic crop in China with strong competitiveness in the global market (Ghosh, 2001; Jiang et al., 2006). Pericarp browning is an intuitive index for evaluating the quality of litchi fruit. Many studies have investigated the causes and mechanisms of pericarp browning in postharvest litchi fruit and methods for its prevention. Water loss (Jiang & Fu, 1999), pathogen infection (Wu et al., 2017), enzymatic catalysis by PPO (Sun et al., 2008), POD (Jiang, Duan, Joyce, Zhang, & Li, 2004) and laccase (Fang et al., 2015) have been identified as the main factors inducing pericarp browning. Treatment with chemicals containing antioxidants (Ali, Khan, Malik, Nawaz, & Shahid, 2019) and acids (Shah, Khan, & Ali, 2017; Zheng & Tian, 2006), heat water and steam (Kessy, Hu, Zhao, & Zhou, 2016; Olesen, Nacey, Wiltshire, & O'Brien, 2004), coating treatment (Hojo, Durigan, & Hojo, 2011), low‐temperature storage (Khan, Ahmad, Malik, & Amjad, 2012), and controlled atmosphere storage (Ali, Khan, Malik, & Shahid, 2016) effectively retard pericarp browning. In addition to pericarp browning, pulp deterioration decreases the quality of litchi fruits after harvest. Previous studies have rarely investigated the metabolites of postharvest litchi pulps, which determine both the flavor and interior quality. The main carbohydrates, organic acids, amino acids, and polyphenols during development and maturity of litchi pulp have been identified (Kong et al., 2010; Wu et al., 2016; Zhang et al., 2013).

Metabolomics has been applied to the study of the mechanism through which temperature affects the postharvest quality of fruits. Through comparative metabolomics analyses, Yun et al. (2016) found that litchi pericarp senescence under ambient temperature after pre‐cold storage is likely related to an ABA‐mediated increase in the oxidation of lipids, polyphenols, and anthocyanins. Using liquid chromatography/tandem mass spectrometry (LC/MS/MS) technology, Matsumoto and Ikoma (2012) investigated the sugars, organic acids, and amino acid content in the juice sacs of Satsuma mandarin stored at 5, 10, 20, and 30°C for 14 days. The results showed that the content of amino acids has more variability compared with the sugars and organic acids, and the optimal temperature for minimizing the postharvest changes in the amino acid profiles is 10°C.

Although room‐temperature storage usually leads to a shorter shelf life and a high loss of quality, it is currently widely used during the short‐distance transportation and sales of litchi fruits due to its low cost. Low‐temperature storage can reduce the respiration rate and postharvest loss of quality and is thus usually used for long‐term storage and long‐distance logistics. Room‐temperature and low‐ temperature storage usually differentially affect the physiological activities of postharvest fruits and might thus lead to distinct changes in metabolic profiles. In this study, the metabolites in the litchi pulp at harvest, after storage at room temperature for 8 d and after storage at low temperature for 28 d, were determined through ultra‐high‐performance liquid chromatography–electrospray ionization–quadrupole time‐of‐flight mass spectrometry (UHPLC‐ESI‐QTOF‐MS/MS). We then compared the changed metabolites and pathways in litchi pulps after 8 d of storage at room temperature with those in litchi pulps after 28 d of storage at low temperature.

## 2 MATERIALS AND METHODS

### 2.1 Chemicals and reagents

Packaging materials: 0.01‐mm‐thick polyethylene (PE) film with a polyethylene terephthalate (PET) salver was used for room‐temperature storage (25°C ± 1°C), while 0.03‐mm‐thick PE bags were used for low‐temperature storage (5°C ± 1°C). AR‐grade NaOH was purchased from Kermel (Tianjin, China). HPLC‐grade ammonium acetate (NH_4_Ac), ammonium hydroxide (NH_4_OH), ammonium fluoride (NH_4_F), and formic acid (FA) were purchased from Sigma‐Aldrich. HPLC‐grade acetonitrile was purchased from Merck.

### 2.2 Plant materials, treatments, and sampling

Commercial mature litchi (*Litchi chinensis* Sonn. cv. “Feizixiao”) fruits with a partially red pericarp (1/3 to 2/3 of the entire pericarp area) were harvested from an orchard at Hualong Fruit & Vegetable Freshness Co., Ltd, on June 15, 2017. The litchi trees were grown using commercial standard practices for controlling both disease and insects. The harvested fruits were immediately transported to the laboratory. More than 600 litchi fruits without any disease or damage were selected and dipped in solution (0.5 g of prochloraz and 1 g of iprodione per liter of water) for 2 min. The fruits were then dried at room temperature for 10 min and used for the storage treatments. Half of the fruits were placed into PET salvers (approximately 20 fruits per salver), packed in 0.01‐mm‐thick PE films, and stored at room temperature with 85% relative humidity. The other half of the fruits were packed in 0.03‐mm‐thick PE bags (20 fruits per bag) and stored at low temperature with 85% relative humidity. Litchi fruits stored at room temperature for 0, 2, 4, 6, and 8 d and litchi fruits stored at low temperature for 0, 7, 14, 21, and 28 d were used to determine the respiration rate, total soluble solid (TSS) content, and titratable acid (TA) content.

Litchi fruits were sampled at harvest (0 d), after storage at room temperature for 8 d (RT‐8 d) and after storage at low temperature for 28 d (LT‐28 d). The RT‐8 d and LT‐28 d fruits showed no severe pericarp browning (pericarp browning <3.0), mildew, or rot. Six biological repeats of each sample were measured, rapidly frozen in liquid nitrogen, and stored at −80°C until use for UHPLC‐ESI‐QTOF‐MS/MS analysis.

### 2.3 Measurement of the respiration rate

Twenty fruits were randomly selected and placed in a hermetically sealed box for 2 hr at room temperature or low temperature. The CO_2_ concentration in the box was determined using a gas chromatograph (6820, Agilent Technologies) equipped with a 30‐meter chromatographic column (HP‐PLOT‐Q). The conditions were set as follows: column temperature, 30°C; carrier gas, 99.999% N_2_; thermal conductivity detector (TCD) temperature, 250°C. The respiration rate after room‐temperature or low‐temperature storage for the various durations was measured in triplicate samples and calculated according to equation:

$$\text{Respiration}\;\;\text{rate}\;\left(\text{mg}\;\;{\text{kg}}^{-1}\;\;h^{-1}\right)=\frac{A\times \left(V_{1}-V_{2}\right)\times M\times 273}{H\times W\times 22.4\times \left(273+T\right)}$$

A: CO_2_ concentration (%); V_1_: volume (ml) of the hermetically sealed box; V_2_: the total volume (ml) of the fruits; M: the molar mass of CO_2_; H: the time in the sealed environment (hr); W: the total fruit weight (kg); T: the storage temperature (Zhu, Li, Yuan, Chen, & Chen, 2010).

### 2.4 Quality analysis

The pulp was separated and used for juicing and determination of the TSS content using a Brix refractometer (PAL‐1, ATAGO Co., Ltd.) and the TA content according to GB 5009.239–2016 (National Standard for Determination of Food Acidity). The quality of three biological replicates was analyzed at each time point.

### 2.5 UHPLC‐ESI‐QTOF‐MS/MS analysis

Pulp samples of 0 d, RT‐8 d, and LT‐28 d litchi fruits were ground into powder in liquid nitrogen. A 60 mg sample of powder was added to 1 ml of precooled methyl alcohol/acetonitrile/water (2:2:1, v/v) and adequately vortexed. After ultrasonic decomposition in an ice bath for 30 min, the samples were incubated for 1 hr at −20°C to precipitate the protein and then centrifuged at 15,700*g* and 4°C for 15 min. The supernatants were collected, dried under vacuum, and stored at −80°C. The samples were redissolved in 100 μl of acetonitrile/water (1:1, v/v), adequately vortexed, and then centrifuged at 18,200*g* and 4°C for 15 min. The supernatant was filtered with a 0.22‐μm syringe nylon filter prior to LC‐MS/MS analysis.

The analyses were performed using an UHPLC (1290 Infinity LC, Agilent Technologies) equipped with a HILIC column (ACQUITY UPLC BEH Amide, 2.1 mm × 100 mm, 1.7 μm, Waters) and coupled to an electrospray ionization–quadrupole time‐of‐flight mass spectrometer (Triple TOF 5600, AB Sciex). The samples were placed in an autosampler at 4°C in a random order. Specifically, for each sample, a volume of 2.0 μl was loaded into the column and maintained at 25°C with a flow rate of 0.3 ml/min. The mobile phase was composed of buffer A (25 mM NH_4_Ac and 25 mM NH_4_OH in water) and buffer B (100% acetonitrile). The gradient of the mobile phase was programmed as follows: 95% B for 1 min and was linearly reduced to 65% in 13 min, and then was reduced to 40% in 2 min and kept at 40% for 2 min, and then increased to 95% in 0.1 min, with a 5‐min re‐equilibration period employed. Samples were detected in both the positive and negative ESI modes.

The ESI source conditions were set as follows: Ion Source Gas1 (Gas1), 60; Ion Source Gas2 (Gas2), 60; curtain gas (CUR), 30; source temperature, 600°C; IonSpray Voltage Floating (ISVF), ± 5,500 V. In the MS acquisition, the instrument was set to acquire over an m·z^−1^ range of 60–1000 Da, and the accumulation time for the TOF MS scan was set to 0.20 s/spectra. In the MS/MS acquisition mode, the instrument was set to acquire m·z^‐1^ range of 25–1000 Da, and the accumulation time for the product ion scan was set to 0.05 s/spectra. The product ion scan was acquired through information‐dependent acquisition (IDA) under the high sensitivity mode. The collision energy (CE) was fixed to 35 V with ± 15 eV. The declustering potential (DP) was set to ± 60 V.

The raw MS data (wiff.scan files) were converted to MzXML files using ProteoWizard MS Convert and processed using XCMS, and this processing included feature detection, retention time correction, and alignment. The metabolites were identified based on mass accuracy (<25 ppm) and the MS/MS data and then matched with our database of standards.

### 2.6 Statistical analysis

In the extracted ion features, only the variables detected more than three repeats (six repeats in total) in at least one sample were retained. SIMCA‐P 14.1 (Umetrics) was used for partial least‐squares‐discriminant analysis (PLS‐DA). The variable influence on projection (VIP) value and *p*‐value of each metabolite were obtained from the PLS‐DA model and through an analysis of the raw data using two‐tailed Student's *t* test. The metabolites with VIP values >1 and *p*‐values <.1 were considered to be differentially expressed metabolites (DEMs) in RT‐8 d (or LT‐28 d) litchi compared with litchi at 0 d.

SigmaPlot software (version 12.3, Systat Software Inc.) and Adobe Illustrator software (CS5, Adobe Systems Inc.) were used for image processing. SPSS software (version 21.0, SPSS Inc.) was used to analyze the least significant difference (LSD) at the 5% level.

## 3 RESULTS AND DISCUSSION

### 3.1 Changes in the appearance and quality of litchi pulp during storage at room temperature and low temperature

**Figure 1:**
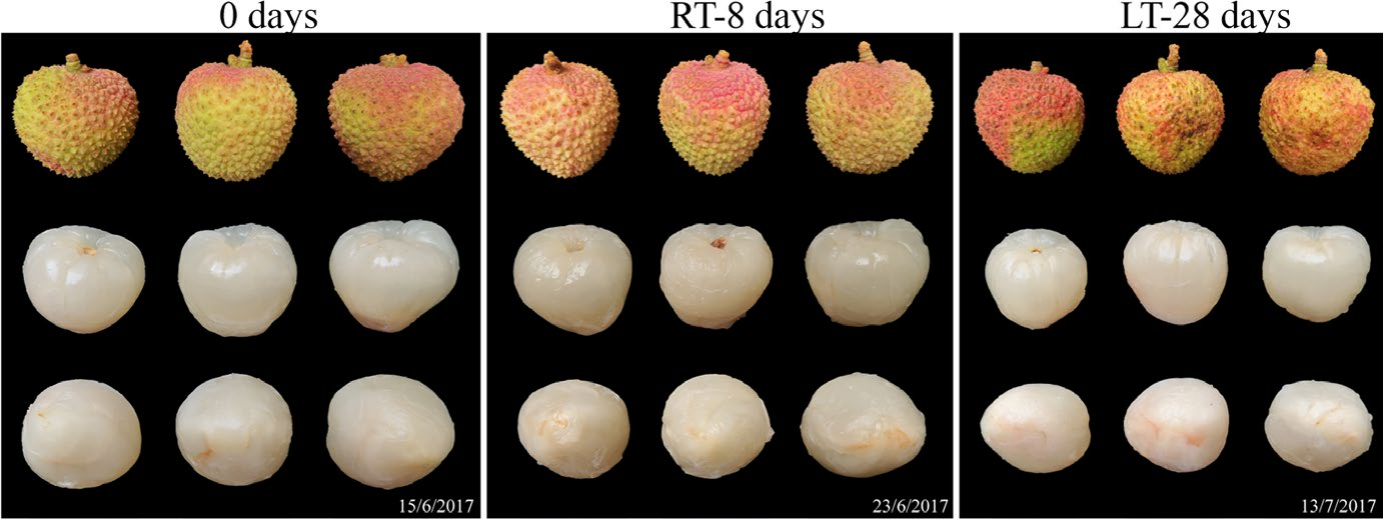
Appearance of the litchi fruits at 0 d, the RT‐8 d, and LT‐28 d litchi fruits. RT‐8 d, litchi pulp after 8 d of storage at room temperature. LT‐28 d, litchi pulp after 28 d of storage at low temperature

The pulps at 0 d were glossy white and translucent, whereas the pedicels and tops of the RT‐8 d and LT‐28 d pulps exhibited slight browning and decreased transparency compared with the pulps at 0 d (Figure 1). The chromatic indices (a*, b*, C*, and h°) revealed no significant difference in colors between the RT‐8 d pulps and the pulps at 0 d; only the L* index of the RT‐8 d pulps was decreased. In contrast, a significant difference in color was observed between the LT‐28 d pulps and the pulps at 0 d (Figure S1).

**Figure 2:**
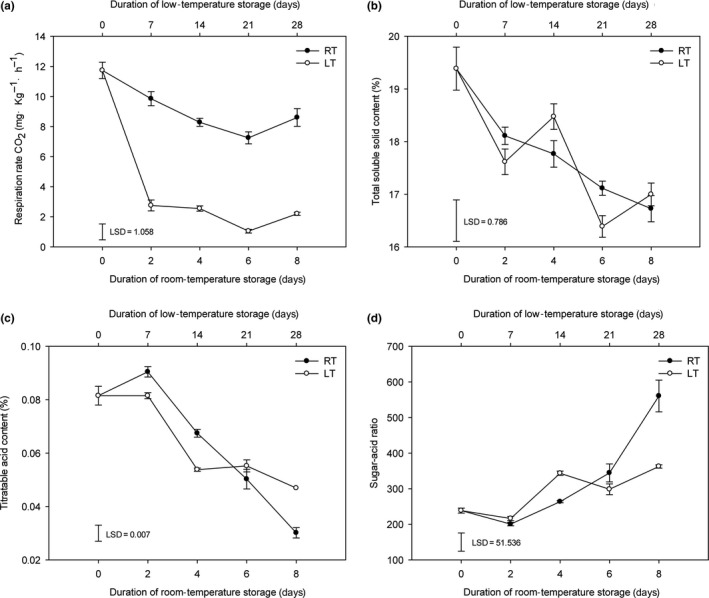
Changes in the (a) respiration rate, (b) total soluble solid content, (c) titratable acid content, and (d) sugar‐acid ratio of litchi fruits during room‐temperature and low‐temperature storage. RT, room temperature. LT, low temperature

The respiration rate of the room‐temperature‐stored fruits decreased from 0 d to 6 d and increased from 6 d to 8 d, whereas that of the low‐temperature‐stored fruits reduced from 0 d to 21 d and increased from 21 d to 28 d. Overall, the respiration rate of the low‐temperature‐stored fruits was significantly lower than that of the room‐temperature‐stored fruits throughout the storage period (Figure 2a), which indicated that low‐temperature storage significantly inhibited the postharvest respiration of litchi fruit. Both the TSS content and TA content in the fruits decreased during room‐temperature‐ and low‐temperature‐stored fruits. The TSS and TA contents of the room‐temperature‐stored fruits exhibited a consistently decreasing trend, whereas those of the low‐temperature‐stored fruits exhibited a fluctuating downward trend during storage. Notably, no significant difference in the TSS content was found between the RT‐8 d and LT‐28 d fruits, whereas the TA content of the LT‐28 d fruits was significantly higher than that of the RT‐8 d fruits (Figure 2b‐c). Moreover, the sugar‐acid ratio is an important index in the evaluation of fruit flavor. The sugar‐acid ratio of the RT‐8 d fruits was significantly higher than that of the LT‐28 d fruits (Figure 2d). The different trends found for the respiration rate, TSS content, and TA content indicated differences in both metabolites and pathways between the room‐temperature‐ and low‐temperature‐stored fruits. Sensory evaluations revealed that all indices of the LT‐28 d fruits showed significant deterioration compared with the fruits at 0 d and the RT‐8 d fruits. The RT‐8 d fruits exhibited significant deterioration compared with the fruits at 0 d in terms of both flavor and overall taste indices (Table S1). Previous studies have shown that the sweetness, acidity, and flavor of fruits are mainly influenced by the types and levels of carbohydrates, organic acids, and amino acids (Keutgen & Pawelzik, 2008; Malundo, Shewfelt, & Scott, 1995). However, the above‐mentioned data are insufficient to explain the changes in pulp quality, and a metabolomics analysis was thus performed to interpret the metabolite changes.

### 3.2 DEMs in RT‐8 d and LT‐28 d fruits compared with fruits at 0 d

**Figure 3:**
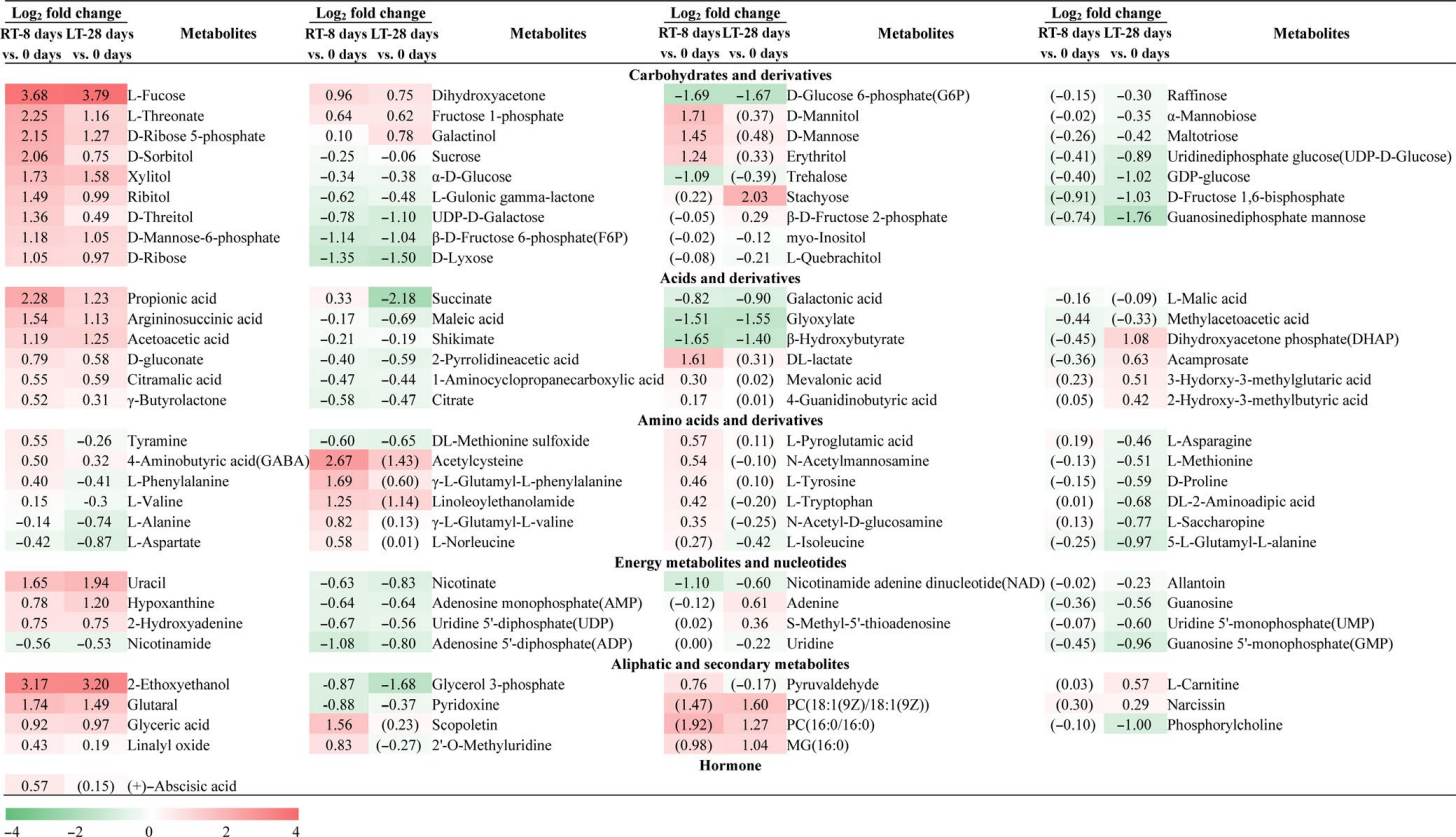
Differentially expressed metabolites in the RT‐8 d and LT‐28 d pulps compared with the pulps at 0 d. The values in brackets indicate nonsignificant differences in the levels of these metabolites in the RT‐8 d or LT‐28 d pulps compared with the pulps at 0 d. RT‐8 d versus 0 d, RT‐8 d pulps compared with the pulps at 0 d. LT‐28 d versus 0 d, LT‐28 d pulps compared with the pulps at 0 d

To analyze the fundamental metabolic processes in fruits related to room‐temperature and low‐temperature storage, the metabolites of the pulps at 0 d, RT‐8 d pulps, and LT‐28 d pulps were analyzed by UHPLC‐ESI‐QTOF‐MS/MS. Nineteen carbohydrates and derivatives, fifteen acids and derivatives, seven amino acids and derivatives, nine energy metabolites and nucleotides, and six aliphatic and secondary metabolites were identified as common DEMs in the RT‐8 d and LT‐28 d pulps compared with the pulps at 0 d. Four carbohydrates and derivatives, five acids and derivatives, ten amino acids and derivatives, three aliphatic and secondary metabolites, and one hormone were identified as unique DEMs in the RT‐8 d pulps compared with the pulps at 0 d. Eleven carbohydrates and derivatives, four acids and derivatives, seven amino acids and derivatives, seven energy metabolites and nucleotides, and six aliphatic and secondary metabolites were identified as unique DEMs in the LT‐28 d pulps compared with the pulps at 0 d (Figure 3).

Based on the above‐described results, the numbers of organic acids and amino acids among the DEMs in the RT‐8 d pulps compared with the pulps at 0 d were higher than those among the DEMs in the LT‐28 d pulps compared with the pulps at 0 d. In contrast, the numbers of carbohydrates, nucleotides, and aliphatic and secondary metabolites among the DEMs in the LT‐28 d pulps compared with the pulps at 0 d were higher than those among the DEMs in the RT‐8 d pulps compared with the pulps at 0 d. With the exception of the abundances of succinate, tyramine, L‐phenylalanine, and L‐valine, which were increased in the RT‐8 d pulps compared with the pulps at 0 d and decreased in the LT‐28 d pulps compared with the pulps at 0 d, the expression trends observed for the other DEMs in the RT‐8 d and LT‐28 d pulps compared with the pulps at 0 d were consistent. The unique DEMs in the RT‐8 d or LT‐28 d pulps compared with the pulps at 0 d belong to different metabolic pathways.

Carbohydrates are important respiratory substrates and osmotic regulators (Hellebusi, 1976; Tarczynski, Jensen, & Bohnert, 1993). Compared with the pulps at 0 d, the levels of sucrose and α‐D‐glucose were decreased in both RT‐8 d and LT‐28 d pulps, whereas those of L‐fucose, D‐sorbitol, xylitol, and galactinol were increased. In particular, the accumulation of D‐sorbitol and D‐mannose is important for osmotic regulation (Hasegawa, Bressan, Zhu, & Bohnert, 2000). With the exception of trehalose, the abundances of all unique differentially expressed carbohydrates were increased only in the RT‐8 d pulps compared with the pulps at 0 d. In addition, the abundances of all unique differentially expressed carbohydrates, with the exception of stachyose and β‐D‐fructose 2‐phosphate, were decreased only in the LT‐28 d pulps compared with the pulps at 0 d. The variations observed in the levels of some carbohydrates were quite different between the RT‐8 d and LT‐28 d pulps compared with the pulps at 0 d, for example, the fold changes in the levels of L‐threonate, D‐ribose 5‐phosphate, D‐sorbitol, and D‐threitol (all of which are low‐abundance carbohydrates) in the RT‐8 d compared with the pulps at 0 d was greater than those of the LT‐28 d pulps compared with the pulps at 0 d, whereas the levels of sucrose and α‐D‐glucose, which are high‐abundance carbohydrates, changed only slightly. Sucrose, glucose, and fructose are the main carbohydrate constituents in litchi pulp (Wu et al., 2016). These results indicate that sweetness is mainly influenced by decreased levels of high‐abundance carbohydrates such as sucrose and glucose, and that changes in the levels of low‐abundance carbohydrates are secondary factors. These findings are consistent with the measured TSS content (Figure 2b).

The L‐malic acid levels were decreased in the RT‐8 d pulps compared with the pulps at 0 d. Succinate, an important substance that participates in the tricarboxylic acid cycle (TCA cycle) and GABA shunt (Bouche, Lacombe, & Fromm, 2003), was found at higher levels in the RT‐8 d pulps compared with the pulps at 0 d and at lower levels in LT‐28 d pulps compared with the pulps at 0 d. Tartaric acid and vitamin C are not DEMs, but we found that the vitamin C content decreased during storage and was lower in the LT‐28 d pulps compared with the RT‐8 d pulps (Figure S2). The variations in the abundances of some organic acids showed notable differences between the RT‐8 d and LT‐28 d pulps compared with the pulps at 0 d, for example, the fold changes in the levels of propionic acid and succinate in the RT‐8 d compared with the pulps at 0 d was greater than those of the LT‐28 d pulps compared with the pulps at 0 d. Propionic acid is present at a low level in litchi pulp, and L‐malic acid, which was found at the highest abundance, was present at significantly reduced levels only in the RT‐8 d pulps. Malic acid, tartaric acid, succinic acid, and ascorbic acid (vitamin C) are the main acid constituents in litchi pulp (Hu, Wang, & Hu, 2005). These results indicate that the changes in acidity are mainly due to the downregulation of high‐abundance organic acids, such as malic acid, succinic acid, and ascorbic acid (vitamin C), and that changes in the levels of low‐abundance organic acids are secondary factors. The organic acid levels in the RT‐8 d pulps were lower than those in the LT‐28 d pulps, which is consistent with the measured TA content in the RT‐8 d pulps (Figure 2c).

4‐Aminobutyric acid (GABA), which is the main amino acid constituent and an important nutritional ingredient of litchi pulp (Bouche et al., 2003; Wu et al., 2016), plays an important role in the resistance to adversity (Bouche et al., 2003; Kinnersley, 2000). GABA was increased in the RT‐8 d and LT‐28 d pulps compared with the pulps at 0 d. Previous studies have shown that low temperature can increase the abundance of GABA in postharvest longan fruit (Zhou, Ndeurumio, Zhao, & Hu, 2016). The levels of the differentially expressed amino acids in the RT‐8 d pulps compared with the pulps at 0 d were increased, and this finding was obtained for all amino acids except L‐alanine, L‐aspartate, and DL‐methionine sulfoxide. The levels of the differentially expressed amino acids in the LT‐28 d pulps compared with the pulps at 0 d were decreased, and this finding was obtained for all amino acids with the exception of GABA. We conjectured that these amino acids might be transformed from carbohydrates and organic acids, which are substrates of respiratory metabolism during short‐term room‐temperature storage or might be associated with protein hydrolysis. Late during low‐temperature storage, the substrates of respiratory metabolism are amino acids, which are finally transformed to aliphatic metabolites and ketone precursors (Nelson & Cox, 2013). Therefore, the abundances of amino acids in the RT‐8 d pulps were higher than those in the LT‐28 d pulps.

With the exception of uracil, hypoxanthine, and 2‐hydroxyadenine, the levels of the common differentially expressed energy metabolites and nucleotides in the RT‐8 d and LT‐28 d pulps compared with the pulps at 0 d were all decreased. No unique differentially expressed energy metabolites and nucleotides were found only in the RT‐8 d pulps compared with the pulps at 0 d, whereas seven unique DEMs were found only in the LT‐28 d pulps compared with the pulps at 0 d. The abundances of all these metabolites, with the exception of adenine and S‐methyl‐5’‐thioadenosine, exhibited decreasing trends. Therefore, we speculated that the balance between the supply and consumption of energy during long‐term storage is unstable, resulting in induction of the degradation of nucleotides and causing disorders related to physiological metabolism. The number of differentially expressed energy metabolites and nucleotides in the LT‐28 d pulps compared with the pulps at 0 d was significantly higher than that in the RT‐8 d pulps compared with the pulps at 0 d, and the abundances of most of these metabolites decreased. This result indicates that the LT‐28 d pulps exhibited more severe energy loss and senescence compared with the RT‐8 d pulps.

The abundances of all differentially expressed aliphatic and secondary metabolites, with the exception of glycerol 3‐phosphate and pyridoxine, were increased both in the RT‐8 d and in the LT‐28 d pulps compared with the pulps at 0 d. These changes might be related to the respiratory metabolism and stress response. In particular, the increases in the unique differentially expressed aliphatic metabolites found only in the LT‐28 d pulps compared with the pulps at 0 d, such as PC(18:1(9Z)/18:1(9Z)), PC(16:0/16:0), and MG(16:0), might be associated with the low temperature stress response (Siminovitch, Rheaume, Pomeroy, & Lepage, 1968). The number of differentially expressed aliphatic and secondary metabolites in the LT‐28 d pulps compared with the pulps at 0 d was significantly greater than that in the RT‐8 d pulps compared with the pulps at 0 d, and these might be associated with an active secondary metabolism. The increased levels of (+)‐abscisic acid in the RT‐8 d pulps compared with the pulps at 0 d exert have an important regulatory effect on fruit resistance and senescence (Yang & Feng, 2015).

### 3.3 Pathways associated with the DEMs in the RT‐8 d and LT‐28 d pulps compared with the pulps at 0 d

The DEMs in the RT‐8 d and LT‐28 d pulps compared with the pulps at 0 d were analyzed using the KEGG database by Fisher's exact test, and the similarities and differences in the main metabolic pathways were compared (Kanehisa, Goto, Sato, Furumichi, & Tanabe, 2012). The metabolic pathways that showed major changes both in the RT‐8 d and in the LT‐28 d pulps compared with the pulps at 0 d were “ABC transporters,” “fructose and mannose metabolism,” “nicotinate and nicotinamide metabolism,” “alanine, aspartate, and glutamate metabolism,” “galactose metabolism,” “aminoacyl‐tRNA biosynthesis,” “amino sugar and nucleotide sugar metabolism,” “propanoate metabolism,” “oxidative phosphorylation,” “pentose phosphate pathway,” “carbon fixation in photosynthetic organisms,” “cyanoamino acid metabolism,” and “pantothenate and CoA biosynthesis.” The main metabolic pathways found only in the RT‐8 d pulps compared with the pulps at 0 d were “phenylalanine, tyrosine, and tryptophan biosynthesis,” “glycine, serine, and threonine metabolism,” “pyruvate metabolism,” “glyoxylate and dicarboxylate metabolism,” “TCA cycle,” “tyrosine metabolism,” and “glycolysis/gluconeogenesis.” The main metabolic pathways found only in the LT‐28 d pulps compared with the pulps at 0 d were “zeatin biosynthesis,” “glycerolipid metabolism,” “purine metabolism,” “cysteine and methionine metabolism,” “pyrimidine metabolism,” “ascorbate and aldarate metabolism,” and “glycerophospholipid metabolism” (Table 1). These findings demonstrated that the respiratory metabolic pathways were notably changed during the storage of litchi fruits at either room temperature or low temperature, which resulted in losses in fruit quality. During this process, the metabolic pathways related to carbohydrates, organic acids, and amino acids might be highly active in RT‐8 d litchi pulps, whereas the metabolic pathways related to aliphatic metabolites and nucleotides might be highly active in LT‐28 d litchi pulps.

**Table 1:** Top 20 KEGG pathways enriched in the differentially expressed metabolites detected in the RT‐8 d and LT‐28 d pulps compared with the pulps at 0 d

Comparison	Pathway description
Identical	ABC transporters
	Fructose and mannose metabolism
	Nicotinate and nicotinamide metabolism
	Galactose metabolism
	Alanine, aspartate, and glutamate metabolism
	Aminoacyl‐tRNA biosynthesis
	Amino sugar and nucleotide sugar metabolism
	Propanoate metabolism
	Oxidative phosphorylation
	Pentose phosphate pathway
	Cyanoamino acid metabolism
	Carbon fixation in photosynthetic organisms
	Pantothenate and CoA biosynthesis
RT−8 d versus 0 d^a^	Phenylalanine, tyrosine, and tryptophan biosynthesis
	Glycine, serine, and threonine metabolism
	Pyruvate metabolism
	Glyoxylate and dicarboxylate metabolism
	TCA cycle
	Tyrosine metabolism
	Glycolysis/gluconeogenesis
LT−28 d versus 0 d^b^	Zeatin biosynthesis
	Glycerolipid metabolism
	Purine metabolism
	Cysteine and methionine metabolism
	Pyrimidine metabolism
	Ascorbate and aldarate metabolism
	Glycerophospholipid metabolism

a RT‐8 d versus 0 d, RT‐8 d pulps compared with the pulps at 0 d.

b LT‐28 d versus 0 d, LT‐28 d pulps compared with the pulps at 0 d.

Fisher's exact test was used to analyze the significance of the differences (*p*‐value < .05).

**Figure 4:**
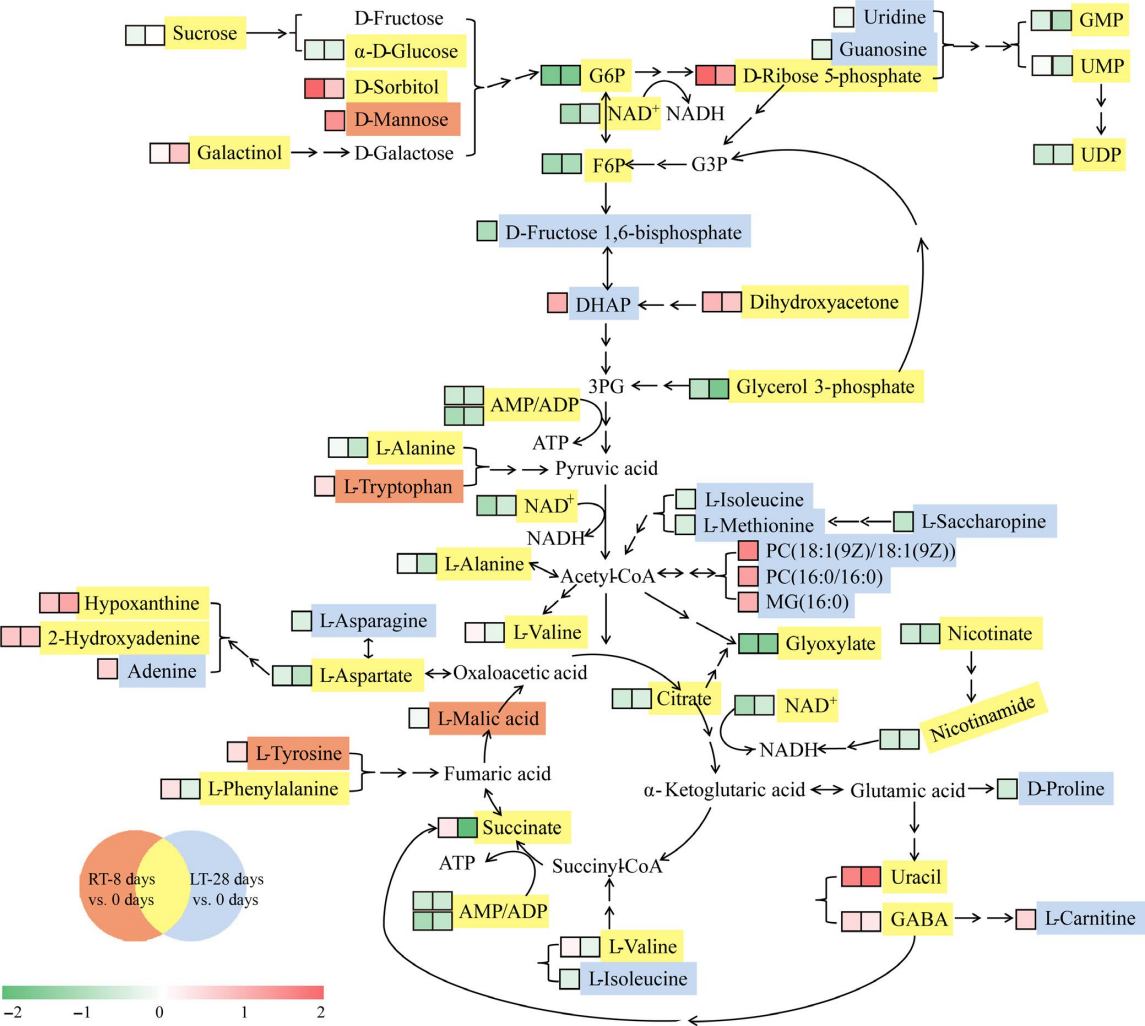
Summary of the significantly changed metabolic pathways during room‐temperature and low‐temperature storage. Yellow background, differentially expressed metabolites (DEMs) both in the RT‐8 d and in the LT‐28 d pulps compared with the pulps at 0 d; orange background, unique DEMs found only in the RT‐8 d pulps compared with the pulps at 0 d; and blue background, unique DEMs found only in the LT‐28 d pulps compared with the pulps at 0 d. The colored boxes in front of each metabolite indicate its log_2_ (fold change) value based on the heat map shown. The first box shows the log_2_ (RT‐8 d/0 d) value, and the second box shows the log_2_ (LT‐28 d/0 d) value. RT‐8 d versus 0 d, RT‐8 d pulps compared with the pulps at 0 d. LT‐28 d versus 0 d, LT‐28 d pulps compared with the pulps at 0 d

Previous studies have shown that healthy postharvest fruit and vegetable tissues adopt the Embden–Meyerhof pathway (EMP) and the TCA cycle as the trunk pathways for respiratory metabolism and the pentose phosphate pathway (PPP) as the branch pathway for respiratory metabolism. In contrast, the opposite trend is observed in deteriorated tissues (Lin et al., 2016, 2018; Zhang et al., 2017). Respiratory metabolism is an active metabolic pathway that constitutes the trunk pathway. Although low‐temperature storage can delay senescence, inhibit biotic stress, and reduce the respiration rate, the challenges associated with long‐term storage, such as nutrient consumption, adversity stress, and senescence, cannot be neglected. The PPP is the trunk pathway and provides suitable conditions for aliphatic metabolism and nucleic acid metabolism. The comparison of the RT‐8 d pulps and the LT‐28 d pulps in terms of the EMP, TCA cycle, and PPP metabolic pathways (Figure 4) revealed that the levels of all the DEMs participating in the EMP metabolic pathway (sucrose, α‐D‐glucose, D‐sorbitol, D‐mannose, G6P, F6P, D‐fructose 1,6‐bisphosphate, and DHAP), the TCA cycle (citrate, succinate, and L‐malic acid), and the PPP (D‐ribose 5‐phosphate) exhibited notable changes. The differentially expressed carbohydrates, organic acids, and energy metabolites in the RT‐8 d and LT‐28 d pulps, such as sucrose, α‐D‐glucose, G6P, F6P, D‐fructose 1,6‐bisphosphate, DHAP, citrate, L‐malic acid, NAD, AMP, and ADP, were consumed in the EMP‐TCA cycle metabolic pathways. It has been speculated that some of the carbohydrates and organic acids in the RT‐8 d pulps were converted to amino acids during respiration, which caused the increase in the L‐valine and GABA levels observed in the RT‐8 d pulps compared with the pulps at 0 d. Moreover, some of the amino acids (L‐alanine, L‐tryptophan, L‐tyrosine, and L‐phenylalanine) were converted to carbohydrates and organic acids, and participated in the EMP‐TCA cycle metabolic pathways again to ensure maintenance of the respiratory metabolism. In contrast, the levels of those differentially expressed carbohydrates, organic acids, amino acids, and nucleotides in the LT‐28 d pulps compared with the pulps at 0 d were decreased. It is thus possible that the differentially expressed amino acids, such as L‐isoleucine, L‐methionine, L‐saccharopine, and L‐alanine, were converted to aliphatic metabolites (PC and MG) via acetyl‐CoA, and this phenomenon might be related to changes in the membrane lipid composition in response to low temperature stress.

## 4 CONCLUSIONS

The differences in the respiration rates, TSS content, and TA content showed that room‐temperature and low‐temperature storage exert different effects on the quality of litchi pulps. An UHPLC‐ESI‐QTOF‐MS/MS analysis was performed to uncover the metabolism underlying these effects. Our results demonstrated that the RT‐8 d and LT‐28 d pulps presented active fructose and mannose metabolism and increased catabolism of nicotinate, nicotinamide, alanine, aspartate, and glutamate. Decreased levels of key metabolites in the EMP‐TCA cycle and an accumulation of phenylalanine, tyrosine, and tryptophan were observed in the RT‐8 d pulps. Interestingly, higher consumption of nucleotide metabolites and the biosynthesis of aliphatic metabolites were found in the LT‐28 d pulps. The above‐described results indicated that active EMP‐TCA cycle pathways in room‐temperature‐stored litchi fruit might be associated with the high respiration rate; moreover, the promotion of the PPP pathway and active aliphatic biosynthesis detected in the LT‐28 d pulps could be related to inhibited respiration and various stresses during long‐term low‐temperature storage. The results definitely provide a metabolic fingerprint that reveals the different effects of room‐temperature and low‐temperature storage on the physiological activities and quality of litchi pulp.

## CONFLICT OF INTEREST

The authors declare that they do not have any conflict of interest.

## ETHICAL APPROVAL

This study does not involve any human or animal testing.

## INFORMED CONSENT

Written informed consent was obtained from all study participants.
