# Supplementary material for: Analysis of metabolomics associated with quality differences between room‐temperature‐ and low‐temperature‐stored litchi pulps
Source: Food Sci Nutr. 2019 Sep 30;7(11):3560–9. doi: 10.1002/fsn3.1208 (PMC6848819; doi:10.1002/fsn3.1208)
Supplement: Supplementary file 1 [file FSN3-7-3560-s001.pdf]

Table S1. Differences in sensory properties among the litchi pulps at 0 d, the RT-8 d and LT-28 d litchi pulps. *The different lowercase letters show significant differences at *p*-value < 0.05, as determined by Duncan's multiple range tests. †RT-8 d, litchi pulp after 8 days of storage at room temperature. ‡LT-28 d, litchi pulp after 28 days of storage at low temperature.

Sensory properties	0 d	RT-8 d	LT-28 d
Pulp appearance	6.17±0.44 a	5.67±0.67 ab	3.67±0.67 b
Pulp aroma	5.39±0.47 a	4.67±0.33 a	2.33±0.67 b
Sweetness and acidities	5.50±0.86 a	4.33±0.67 a	1.67±0.33 b
Flavour	6.89±0.53 a	5.33±0.33 b	2.67±0.33 c
Texture	7.00±0.33 a	5.67±0.67 a	3.33±0.33 b
Overall taste	6.33±0.42 a	4.67±0.33 b	2.67±0.33 c

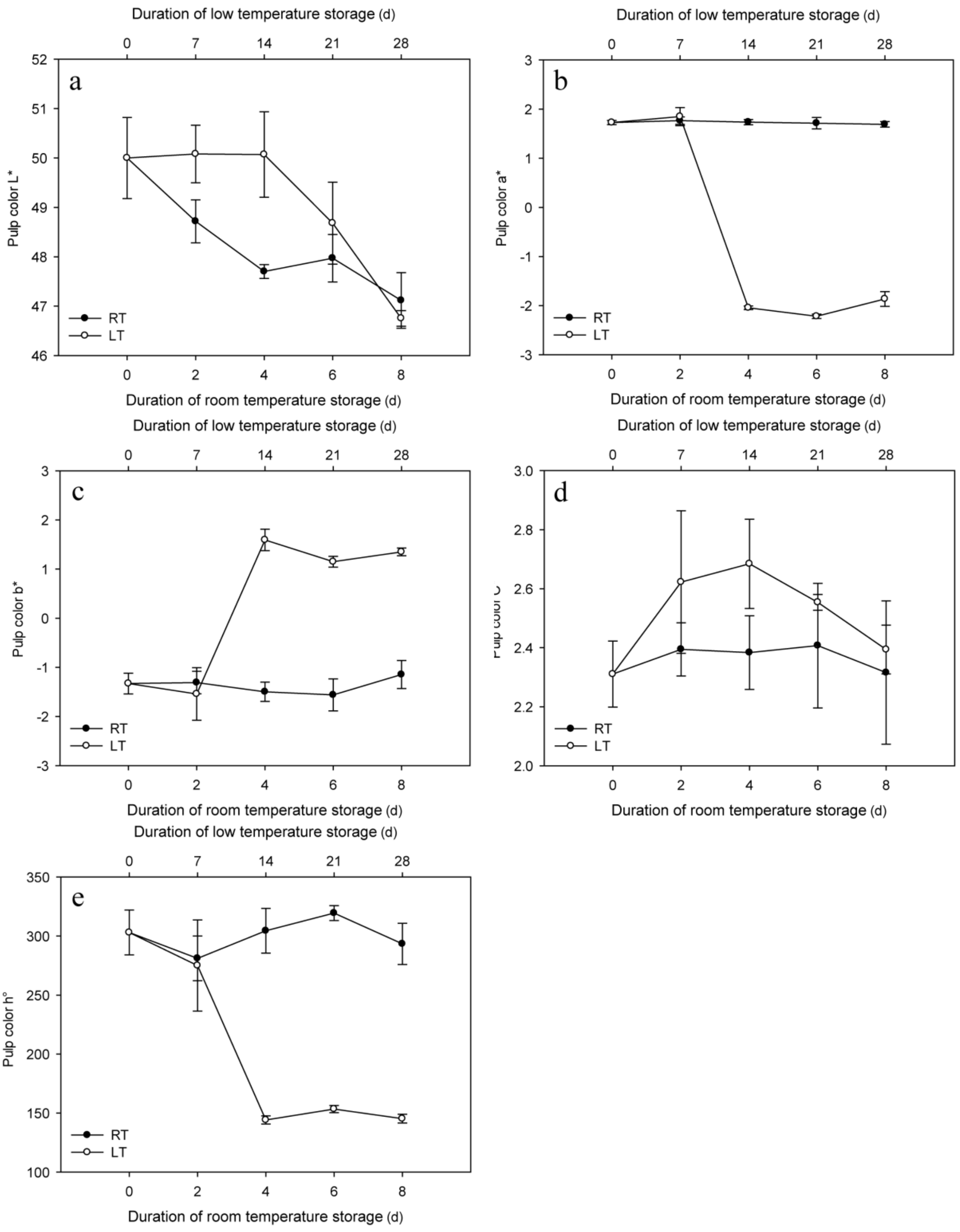

Figure S1. Differences in colour indices between room temperature and low temperature stored litchi pulps. †RT, room temperature. ‡LT, low temperature.

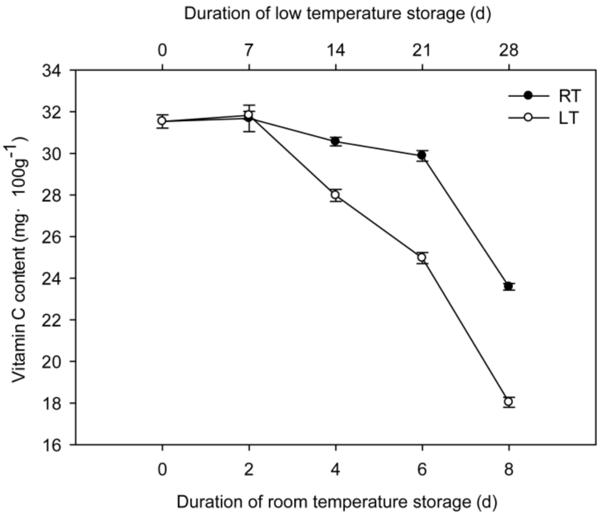

Figure S2. Differences in the vitamin C content between room temperature and low temperature stored litchi pulps. †RT, room temperature. ‡LT, low temperature.
